# Local and regional heterogeneity underlying hippocampal modulation of cognition and mood

**DOI:** 10.3389/fnbeh.2014.00147

**Published:** 2014-05-06

**Authors:** Lindsay Tannenholz, Jessica C. Jimenez, Mazen A. Kheirbek

**Affiliations:** ^1^Department of Pharmacology, Columbia UniversityNew York, NY, USA; ^2^Division of Integrative Neuroscience, New York State Psychiatric InstituteNew York, NY, USA; ^3^Department of Neuroscience, Columbia UniversityNew York, NY, USA; ^4^Department of Psychiatry, Columbia UniversityNew York, NY, USA

**Keywords:** hippocampus, dentate gyrus, adult neurogenesis, anxiety

## Abstract

While the hippocampus has been classically studied for its role in learning and memory, there is significant support for a role of the HPC in regulating emotional behavior. Emerging research suggests these functions may be segregated along the dorsoventral axis of the HPC. In addition to this regional heterogeneity, within the HPC, the dentate gyrus is one of two areas in the adult brain where stem cells continuously give rise to new neurons. This process can influence and be modulated by the emotional state of the animal, suggesting that adult neurogenesis within the DG may contribute to psychiatric disorders and cognitive abilities. Yet, the exact mechanism by which these newborn neurons influence behavior remains unknown. Here, we will examine the contribution of hippocampal neurogenesis to the output of the HPC, and suggest that the role of neurogenesis may vary along the DV axis. Next, we will review literature indicating that anatomical connectivity varies along the DV axis of the HPC, and that this underlies the functional segregation along this axis. This analysis will allow us to synthesize novel hypotheses for the differential contribution of the HPC to cognition and mood.

## Introduction

The hippocampus (HPC) has classically been implicated in learning and memory beginning with studies of Henry Molaison who exhibited profound memory deficits after undergoing a bilateral medial temporal lobectomy to alleviate his drug-resistant seizures (Scoville and Milner, 1957). In recent years, a role for the HPC in emotional control has become more widely appreciated as well. For example, decreased hippocampal volume has been reported in depressed patients (Sheline et al., 1999; Videbech and Ravnkilde, 2004). While it is believed that the HPC plays a critical role in both cognition and mood, the detailed circuitry by which it modulates such seemingly disparate processes remains unclear. Circuit tracing studies show that the output of the HPC differs along the dorsoventral (DV) axis, indicating that depending on the locus of hippocampal output, different downstream structures may be recruited (Fanselow and Dong, 2010). Elucidating how the HPC modulates these targets may provide an entry point for understanding how the HPC regulates both learning and emotion. In addition, local circuit properties of the HPC may influence its net output. Here, we will examine one such property specific to the dentate gyrus (DG) subregion of the HPC: adult hippocampal neurogenesis (AHN). AHN is a unique form of plasticity that is often implicated in cognitive function and anxiety-like behavior. Next, we will explore the HPC’s various outputs particularly focusing on the role of the HPC in anxiety modulation and the candidate regions that may be critical for this modulation. And finally, we will discuss how AHN may modulate local circuitry impacting hippocampal output to downstream targets.

## Local heterogeneity in the dentate gyrus

### Adult neurogenesis

Neural progenitor cells in the subgranular zone of the DG produce new neurons throughout adulthood. In rodents, these adult-born granule cells (abGCs) develop over a period of several weeks during which time they exhibit distinct properties that set them apart from mature GCs (Zhao et al., 2008; Deng et al., 2010; Drew et al., 2013). During their development, GCs exhibit a period of increased synaptic plasticity characterized by a reduced threshold for induction of long-term potentiation (LTP) and an increase in LTP amplitude (Schmidt-Hieber et al., 2004; Ge et al., 2007). By 6–8 weeks after birth, new neurons’ synaptic plasticity becomes indistinguishable from other fully mature GCs (Ge et al., 2007). In humans, recent studies using radiocarbon dating techniques have found that neurogenesis occurs at significant levels in the HPC throughout adulthood (Spalding et al., 2013). Specifically, it was found that the majority of DG cells are subject to exchange, with about 1400 new GCs added to the adult human DG daily, corresponding to an annual turnover rate of 1.75%. This was a striking finding, as it suggested that levels of neurogenesis in humans are comparable to that in mice.

The process of neurogenesis is highly regulated by the cognitive and emotional state of an animal. Interventions such as learning, environmental enrichment (EE), exercise, and antidepressant (AD) treatment increase levels of AHN while negative interventions like chronic stress, increased glucocorticoid levels, and social isolation, lead to decreases (Gould et al., 2000; Malberg et al., 2000; Dranovsky and Hen, 2006). In addition, AHN is required for some of the behavioral effects of ADs (Santarelli et al., 2003; David et al., 2009).

In recent years, the mechanism by which AHN may regulate stress responses has become clearer. Schloesser et al. (2009) showed that mice with suppressed neurogenesis exhibited an increased corticosterone response after exposure to acute restraint stress. In addition, neuroendocrine and behavioral responses to a mild stressor are exaggerated in mice with reduced neurogenesis, linking HPC output with the hypothalamic-pituitary-adrenal (HPA) axis (Snyder et al., 2011). Chronic stress produces a dysregulation in HPC-HPA connectivity, which can be corrected by AD treatment. However, suppressing levels of AHN blunts this restorative ability of fluoxetine (Surget et al., 2011).

In the cognitive realm, it has been proposed that newborn neurons play an essential role in pattern separation, the process of transforming similar inputs into non-overlapping, dissimilar outputs that has been attributed to the DG (Kheirbek et al., 2012a). In a contextual fear discrimination (CFD) paradigm used to test behavioral pattern separation (McHugh et al., 2007), mice whose abGCs had been suppressed by either irradiation (Sahay et al., 2011), genetic means (Tronel et al., 2012), or had been manipulated to reduce the newborn neurons’ enhanced plasticity (Kheirbek et al., 2012b), produced deficits in discriminating between highly similar contexts. Furthermore, using two spatial separation pattern separation tasks, the radial arm maze and a nose-poke touch screen task, Clelland et al. (2009) showed that irradiated mice performed worse than sham mice in conditions of high spatial similarity. Finally, increasing the number of abGCs in the DG improves the animal’s ability to perform context and spatial pattern separation, either by specifically inhibiting their apoptosis (Sahay et al., 2011), or elevating levels of neurogenesis through running (Creer et al., 2010). While these experiments show the importance of abGCs in cognitive tasks, impairments in pattern separation may also contribute to anxiety disorders by impairing memory generalization. Such impairments could underlie the pathological fear responses seen in anxiety disorders such as post-traumatic stress disorder and panic disorder (Kheirbek et al., 2012a). As neurogenesis is highly regulated by an individual’s emotional state, reduced neurogenesis due to stress either before or after a traumatic event could result in a deficit in pattern separation leading to overgeneralization of fear to neutral contexts.

### Dorsoventral gradients in adult neurogenesis

Studies targeting the DG GCs have supported a functional heterogeneity within the structure, which may underlie the DG’s ability to regulate both mood and cognition. In an optogenetic study, it was found that the dorsal DG (dDG) controls exploratory drive and encoding of contextual fear memories while the ventral DG (vDG) regulates innate anxiety (Kheirbek et al., 2013). In addition, studies have shown projections from the entorhinal cortex innervate the DG in a topographic manner (Moser and Moser, 1998) and increased basal network activity in the dDG as measured by immediate early gene (IEG) induction (Piatti et al., 2011). Neuromodulatory influence on the HPC may differ as well, as serotonergic input is enriched in the vDG with a concomitant increase in 5-HT1A receptor mRNA levels (Gage and Thompson, 1980; Tanaka et al., 2012). This suggests that the dorsal and ventral DG may represent distinct neurogenic environments (Piatti et al., 2011). Therefore, the subpopulations that make up the pool of newborn neurons in each region may be differentially effected by environmental and chemical interventions and functionally distinct as well (Kheirbek and Hen, 2011; Samuels and Hen, 2011). Under baseline conditions, the dDG has a higher density of immature neurons and a faster maturation rate (Snyder et al., 2009; Jinno, 2011b; Snyder et al., 2011). Neurogenesis also declines with age but this reduction occurs faster in the vDG (Jinno, 2011a). In addition to baseline differences, stress and ADs have a more prominent influence on neurogenesis in the ventral HPC (vHPC). Stressed mice exhibit decreased cell proliferation and neurogenesis in the vDG, which is reversed by chronic AD treatment (Jayatissa et al., 2006; Tanti et al., 2012). Agomelatine, a melatonin receptor agonist and 5-HT2C receptor antagonist with antidepressant efficacy in humans increased neurogenesis specifically in the vDG of rats, consistent with an enrichment of 5-HT2C in the vHPC (Banasr et al., 2006; Tanaka et al., 2012). In major depressive disorder patients, AD-induced increases in neurogenesis are localized to the anterior HPC (human equivalent of the vHPC in rodents) (Boldrini et al., 2009). Finally, local ablation of neurogenesis in the dorsal or ventral DG confirmed this functional dissociation, with dorsal abGCs being required for CFD, and ventral abGCs being required for the anxiolytic/antidepressant effects of fluoxetine.

Considering the impact of neurogenesis on mood raises the question of how changes within the DG can influence downstream circuitry relevant for stress and anxiety. Identifying the targets of the HPC that are important in mood and emotional processing, and elucidating how the HPC modulates that circuitry, will be essential for understanding the functional role of the HPC in anxiety modulation.

## Regional heterogeneity in the hippocampus

### Hippocampal Circuitry

**Figure 1:**
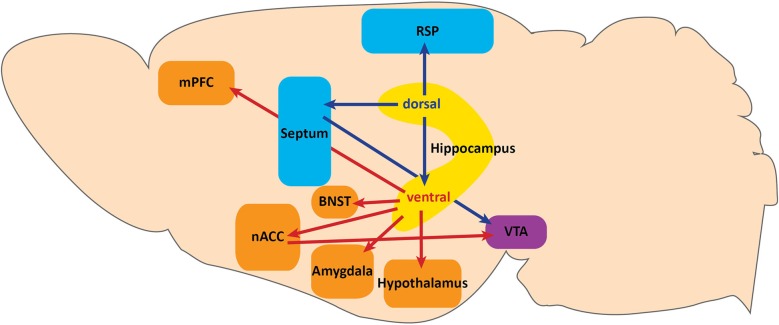
**Projections along the dorsoventral axis of the hippocampus**. The dorsal hippocampus projects to the retrosplenial area (RSP) of the anterior cingulate cortex and to the ventral tegmental area (VTA) via the septum. These projections serve a functional role in context-dependent cognitive processes. Dorsal hippocampus also sends projections to the ventral hippocampus. Projections from the ventral hippocampus include: the medial prefrontal cortex (mPFC), hypothalamus, amygdala, bed nuclues of the stria terminalis (BNST), and the VTA via the nucleus accumbens (nACC). Ventral hippocampal projections function to modulate fear expression and anxiety.

In the tri-synaptic circuit, the entorhinal cortex sends information from association cortices via the perforant path to the DG. DG GCs then send excitatory mossy fiber projections to CA3 pyramidal neurons which project to CA1 via the Schaffer collaterals, and CA1 sends projections to the subiculum. Of these subfields, CA3, CA1, and subiculum can send projections outside of the HPC. It is these projections that may have differing effects on behavior, as dorsal hippocampal targets are primarily involved in spatial memory tasks and context-reward associations (Cenquizca and Swanson, 2007; Luo et al., 2011), while vHPC targets impact emotional expression (Figure 1).

The dorsal HPC (dHPC) may directly impact spatial memory tasks by providing contextual information via dorsal CA1 projections to the retrospenial area of the anterior cingulate cortex (Figure 1; Cenquizca and Swanson, 2007). Indeed, dHPC and retrosplenial lesions have been shown to disrupt learning in spatial memory tasks (Moser et al., 1995; Vann and Aggleton, 2002, 2004; Pothuizen et al., 2004), and dorsal CA1 silencing abolishes behaviorally induced IEG expression in the retrosplenial cortex during spatial memory tasks (Kubik et al., 2012). In addition to modulating spatial memory, the dHPC may provide contextual information for reward-context association tasks involving dopamine release from the ventral tegmental area (VTA) via dorsal CA3 projections to the septum (Figure 1; Risold and Swanson, 1996). Specifically, Luo et al. (2011) found that dorsal CA3 activity disinhibits dopaminergic VTA neurons by activating long-range inhibitory projections in the lateral septum. Although the behavioral relevance of this circuit has not been demonstrated, this study provides a specific circuit through which dHPC may impact the VTA reward system. Taken together, projections from the dHPC serve a functional role in context-dependent cognitive processes.

In the ventral axis, vCA1 and vSUB are the major output centers of the HPC. Although some anatomical distinctions and differences in terminal densities within sub-nuclei have been reported between vCA1 and vSUB projections (Canteras and Swanson, 1992; McDonald, 1998; Kishi et al., 2000; Cenquizca and Swanson, 2007), the majority of their projection patterns are largely overlapping. It is also important to note that the densest projection from vCA1 is to vSUB, further suggesting a large degree of overlap in information processing (Cenquizca and Swanson, 2007). We will therefore discuss vHPC projections in terms of both vCA1 and vSUB, unless otherwise specified.

### vHPC outputs to the nucleus accumbens and VTA

Although Luo et al. (2011) described a pathway for dHPC modulation of VTA dopamine release, the vHPC has also been proposed to impact the VTA reward system (Figure 1; Legault and Wise, 2001; Lisman and Grace, 2005; Valenti et al., 2011). The vHPC projects directly to the nucleus accumbens (nACC; Christie et al., 1987; Totterdell and Smith, 1989), and vSUB activity is necessary for novelty-evoked and stress-induced VTA dopamine release (Legault and Wise, 2001; Valenti et al., 2011). Understanding whether dorsal and ventral HPC modulation of VTA dopamine release are functionally overlapping pathways or serve behaviorally distinct functions will be important for establishing the role of the HPC in reward and novelty processing.

### vHPC outputs to the medial prefrontal cortex

A major target of the ventral HPC is the medial prefrontal cortex (Figure 1; mPFC; Cenquizca and Swanson, 2007). Although hippocampal projections to the mPFC have been traditionally implicated in cognitive spatial memory tasks and goal-directed behavior (Seamans et al., 1998; Seamans and Yang, 2004; Hok et al., 2005; Burton et al., 2009), vHPC input to the mPFC has been studied more recently for its role in innate anxiety and conditioned fear. Interestingly, vHPC-mPFC synchrony has been shown to increase during anxiogenic tasks (Adhikari et al., 2010), and anxiety-related single unit activity within mPFC is modified by vHPC activity (Adhikari et al., 2011). vHPC input has also been implicated in regulating fear expression through mPFC activity modulation in conditioned fear paradigms. Specifically, Sotres-Bayon et al. (2012) established a differential contribution of vHPC and basolateral amygdala (BLA) inputs to the prelimbic (PL) mPFC in fear expression before and after extinction learning. They found that the BLA drives activity of PL neurons leading to fear expression in conditioned rats, while in fear extinction, vHPC activity suppresses fear expression by decreasing PL neuron activity presumably through activation of local inhibitory PL neurons.

### vHPC outputs to the amygdala and bed nucleus of the stria terminalis

The vHPC also projects to most major subfields of the amygdala (Figure 1; Canteras and Swanson, 1992; McDonald, 1998; Pitkänen et al., 2000; Kishi et al., 2006; Cenquizca and Swanson, 2007). While recent evidence has implicated vHPC-amygdala circuitry in innate anxiety and social behavior (Felix-Ortiz et al., 2013; Felix-Ortiz and Tye, 2014), there is considerable evidence implicating this circuit in conditioned fear. Pharmacologic inhibition, lesion studies, and *in vivo* recordings have suggested a coordinated role between the vHPC and amygdala in both contextual and cued fear expression processes (Corcoran and Maren, 2001; Seidenbecher et al., 2003; Maren and Holt, 2004; Corcoran et al., 2005). Additionally, recent studies have more precisely shown that inactivation of both dorsal and ventral hippocampal pyramidal neurons can impact unique aspects of contextual fear conditioning. Goshen et al. (2011) found that acute dorsal CA1 inactivation resulted in disruption of fear memory acquisition and retrieval, and Zhu et al. (2014) found that vHPC inactivation resulted in disruption of memory consolidation, but not encoding or retrieval. It is possible that these effects are mediated by vHPC projections to the BLA complex, given its role in associative learning. Still, specific manipulations of vHPC terminal fields within the amygdala during both encoding and retrieval have not been performed and will be necessary to clearly elucidate their contribution to contextual learning and fear expression.

Nuclei within the amygdala that receive the densest projections from both vCA1 and vSUB are the posteriormedial cortical and posterior basomedial nucleus, which both receive extensive olfactory input and are heavily interconnected with the hypothalamus (Canteras and Swanson, 1992; McDonald, 1998; Kishi et al., 2006; Cenquizca and Swanson, 2007; Hübner et al., 2014). The posterior basomedial amygdala has recently been suggested to participate in a predator response circuit (Martinez et al., 2011; Gross and Canteras, 2012). In this circuit, vHPC input may provide contextual information necessary for proper behavioral responses to predator cues and contextual conditioning. Though the specific role of the vHPC in this circuit remains to be determined, these studies highlight a connection between the vHPC and multiple fear expression pathways.

The bed nucleus of the stria terminalis (BNST) also receives direct projections from the vHPC (Figure 1; Cullinan et al., 1993), and is heavily interconnected with the amygdala, hypothalamus, and VTA (Dong et al., 2001a,b; Dong and Swanson, 2004a,b, 2006a,b,c; Stamatakis et al., 2014). Recently, a differential function for the BNST in these circuits has been implicated in more specific features of anxiety (Jennings et al., 2013; Kim et al., 2013). Future studies using cell-type and projection specific dissection techniques will elucidate not only the differential contribution of each projection field to behavior, but also how the vHPC modulates local circuits within the amygdala, BNST, and mPFC.

### vHPC outputs to the hypothalamus

Given the role of the HPC-HPA connection in the modulation of emotional state, determining how vHPC projections modulate hypothalamic activity will significantly advance our understanding of mood regulation processes. Both vCA1 and vSUB project extensively to many subnuclei of the hypothalamus, including the anterior hypothalamus (AH), lateral hypothalamus (LH), premamillary, ventromedial, dorsomedial, and mammillary bodies, all of which have been implicated in the expression of defensive behaviors (Figure 1; Canteras et al., 1997; Dielenberg et al., 2001; Cezario et al., 2008). Of these nuclei, the AH and LH receive the densest projection from vCA1 (Canteras and Swanson, 1992; Kishi et al., 2000; Cenquizca and Swanson, 2006). Interestingly, activation of GABAergic inputs to AH from the lateral septum, an extension of the septo-hippocampal axis, has recently been shown to increase corticosterone levels and produce persistent anxiety-like behaviors (Anthony et al., 2014). Although HPC contribution to this pathway remains unknown, this study describes a circuit through which the HPC could modulate HPA responses to stress. Dissecting the differential roles of hypothalamic sub-nuclei in defensive, sexual, and feeding behaviors has thus far been challenging given anatomical limitations. However, recent advances have been made (Lin et al., 2011) with novel cell-type specific targeting strategies to overcome those limitations (Silva et al., 2013; Anthony et al., 2014). Utilizing these approaches will increase our understanding of specific hypothalamic sub-nuclei function and further elucidate the potential role of vHPC input in mood regulation.

## Discussion

Thus far, the experiments described demonstrate the importance of AHN in modulating cognition and mood, suggest that the abGCs’ function may be segregated along the septotemporal axis of the DG, and dissect the various outputs of the HPC that likely underlie the functional segregation of the HPC. However, the question of how newborn neurons modify hippocampal output still remains. One possibility is that these neurons are independent units that excite downstream CA3 neurons with which they make functional synapses as early as 2 weeks of age (Gu et al., 2012). Alternatively, there is evidence that abGCs may modulate activity of the mature population of GCs. *In vivo* recordings in mice show that both irradiation and genetic ablation of neurogenesis increase the magnitude of γ bursts in the DG and lead to greater coordination of single unit activity with these γ bursts (Lacefield et al., 2012). Burghardt et al. (2012) has shown mice with ablated neurogenesis were impaired in a conflict condition of the active place avoidance task, and this impairment was associated with increased IEG expression in the dDG, also suggesting abGCs can modulate overall dentate network activity. Furthermore, using voltage sensitive dye imaging, it was found that mice with increased neurogenesis exhibited a decrease in the activation of the granule cell layer. The opposite was seen after suppression of AHN (Ikrar et al., 2013). The mechanism for this modulation remains unknown, but an attractive candidate would be via modulation of local interneurons that may feed-back on to the mature population, in turn altering output to directly impact behavior. Future studies testing the connection between AHN and vHPC output will be crucial for understanding how the HPC is involved in mood regulation.

## Conflict of interest statement

The authors declare that the research was conducted in the absence of any commercial or financial relationships that could be construed as a potential conflict of interest.
